# MALAT1 long ncRNA promotes gastric cancer metastasis by suppressing *PCDH10*

**DOI:** 10.18632/oncotarget.7281

**Published:** 2016-02-09

**Authors:** Ying Qi, Hong Sain Ooi, Jun Wu, Jian Chen, Xiaoli Zhang, Sheng Tan, Qing Yu, Yuan-Yuan Li, Yani Kang, Hua Li, Zirui Xiong, Tao Zhu, Bingya Liu, Zhifeng Shao, Xiaodong Zhao

**Affiliations:** ^1^ School of Biomedical Engineering, Bio-ID Research Center, State Key Laboratory for Oncogenes and Related Genes, Shanghai Jiao Tong University, Shanghai, China; ^2^ Department of Biomedicine, Aarhus University, Aarhus, Denmark; ^3^ Department of Automation, Shanghai Jiao Tong University, Shanghai, China; ^4^ Laboratory of Molecular Tumor Pathology, School of Life Sciences, University of Science and Technology of China, Hefei, Anhui, China; ^5^ Shanghai Center for Bioinformatics Technology, Shanghai, China; ^6^ Shanghai Engineering Research Center of Pharmaceutical Translation, Shanghai, China; ^7^ Department of Surgery, Shanghai Key Laboratory of Gastric Neoplasms, Ruijin Hospital, Shanghai Jiao Tong University School of Medicine, Shanghai, China

**Keywords:** EZH2, RIP-seq, MALAT1, gastric cancer, transcriptional silencing

## Abstract

EZH2, the catalytic component of polycomb repressive complex 2 (PRC2), is frequently overexpressed in human cancers and contributes to tumor initiation and progression, in part through transcriptional silencing of tumor suppressor genes. A number of noncoding RNAs (ncRNAs) recruit EZH2 to specific chromatin loci, where they modulate gene expression. Here, we used RNA immunoprecipitation sequencing (RIP-seq) to profile EZH2-associated transcripts in human gastric cancer cell lines. We identified 8,256 transcripts, including both noncoding and coding transcripts, some of which were derived from cancer-related loci. In particular, we found that long noncoding RNA (lncRNA) MALAT1 binds EZH2, suppresses the tumor suppressor *PCDH10*, and promotes gastric cellular migration and invasion. Our work thus provides a global view of the EZH2-associated transcriptome and offers new insight into the function of EZH2 in gastric tumorigenesis.

## INTRODUCTION

Polycomb group (PcG) proteins are transcriptional repressors that modulate chromatin structure and play critical roles in development and tumorigenesis [1]. Two distinct polycomb repressive complexes (PRC1 and PRC2) have been identified. PRC2 participates in the initiation of transcriptional repression and contains EZH2, a subunit that catalyzes trimethylation of histone 3 Lys27 (H3K27me3) [1, 2]. PRC1 functions downstream of PRC2, binding specifically to H3K27me3 and monoubiquitinating Lys119 of histone H2A (H2AK119ub1) to prevent transcriptional elongation and consolidate transcriptional repression [1]. As a core component of PRC2, EZH2 is highly expressed in various cancer types, including lung, colon, stomach, breast and prostate cancers [3, 4]. The oncogenic potential of EZH2 coupled with the fact that widespread epigenetic deregulation is a hallmark of cancer implicates EZH2 as an important driver of cancer development and progression [3].

A key aspect of polycomb repressive complex biology is its recruitment to specific genomic locations. In *Drosophila*, PcG protein complexes are recruited to chromatin for maintenance of the silent state by sequence elements termed polycomb response elements (PREs) [5]. Although some PRE-like elements have recently been identified at limited genomic loci, mammalian PREs have not yet been well defined [6]. The observation of co-localization of JARID2 and PRC2 in the mammalian genome suggests JARID2 could direct PRC2 to specific target genes [7, 8]. Additionally, the RNA-mediated interaction could be involved in guiding PRC2 to specific genomic targets. One of the best characterized examples is the lncRNA HOTAIR, which is transcribed from the *HOXC* locus and represses *HOXD* expression in *trans* by binding EZH2 and SUZ12 [9]. The lncRNAs ANRIL, Xist, and Tsix are also involved in PRC2-mediated transcriptional repression [10, 11]. However, it should be noted that both ncRNA and other RNA species can readily bind PRC2 [12, 13], which complicates our understanding of the functional consequence of the PRC2-RNA interactions. In this study, we performed a transcriptome-wide profiling of EZH2-bound transcripts in human gastric cancer (GC) cell lines with modified RIP-seq and explored how EZH2 is involved in gastric tumorigenesis through its interaction with RNAs.

## RESULTS

### Transcriptome-wide capture of EZH2-associated RNAs with modified RIP-seq

**Figure 1:**
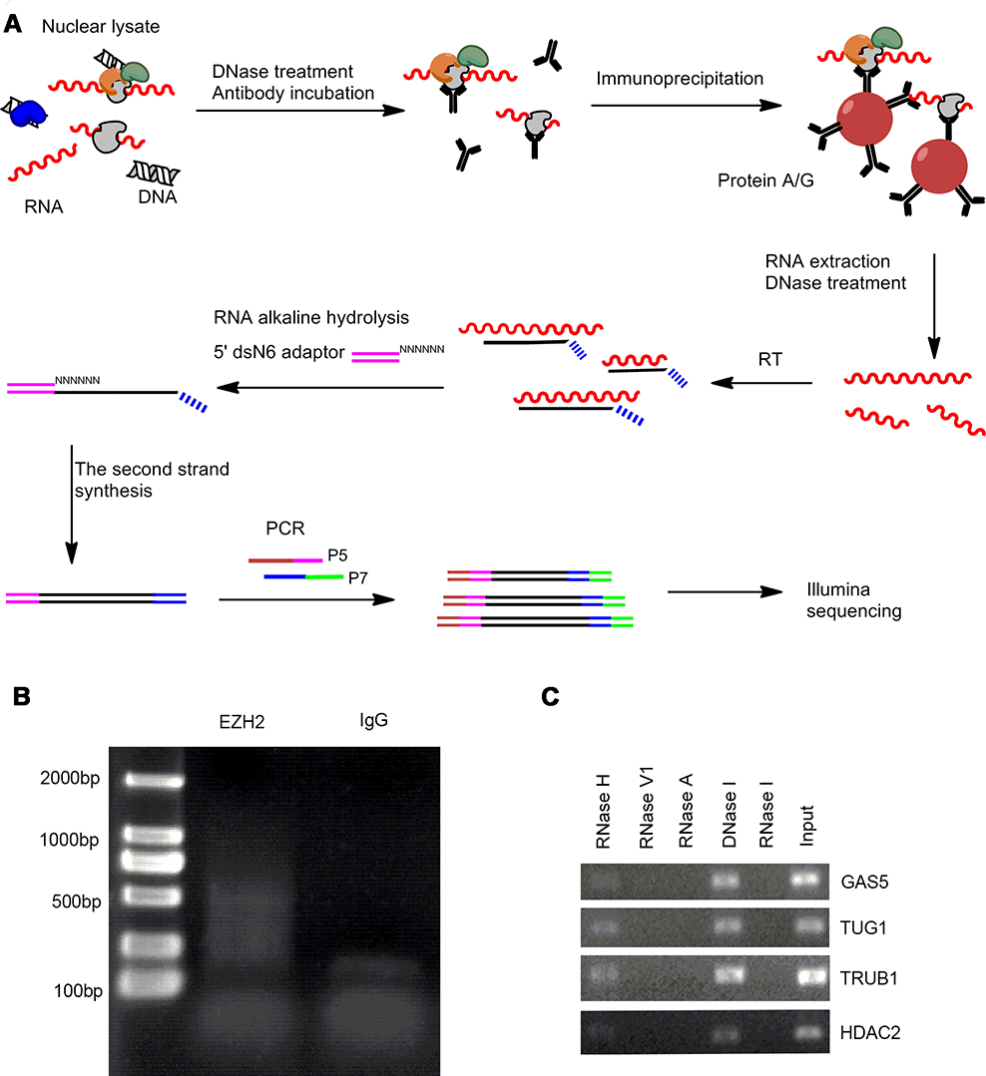
The modified RIP-seq approach for EZH2-interacting RNA profiling analysis (**A**) Schematic representation of modified RIP-seq. (**B**) RIP-PCR products were analyzed in 2% agarose gel. (**C**) Native EZH2-RIP RNAs were treated with different kinds of endoribonuclease, followed by RT-qPCR and agarose gel electrophoresis.

We performed RIP-seq to characterize the EZH2-associated RNAs following a previously described method with some modifications [14]. Briefly, isolated cell nuclei were used for RNA immunoprecipitation with EZH2-specific antibody and the enriched RNAs were reverse transcribed using a random primer flanked with a 7 nucleotide (nt) barcode sequence to track strand information. The second strand cDNA synthesis was performed using the adaptor ligation strategy we reported recently [15]. The resulting RIP libraries were amplified and subjected to Illumina sequencing (Figure 1A).

With this improved RIP-seq approach we performed global profiling of the EZH2-associated RNAs in two GC cell lines MKN45 and AGS and their normal counterpart GES-1. The EZH2 antibody pulldown yielded ∼ 100 ng RNA and generated a cDNA smear larger than 350 nt after PCR amplification (Figure 1B). In contrast, the control IgG pulldown yielded 80% less RNA (∼ 20 ng), suggesting that EZH2 antibody efficiently captures EZH2-interacting RNAs.

To examine the components of the EZH2 pulldown, we treated RIP products with different types of endoribonuclease, including RNase I/RNase A, RNase V1, and RNase H, as well as the deoxyribonuclease DNase I. The endonucleases specifically hydrolyze single-strand RNA, double-strand RNA, RNA in RNA-DNA hybrids, single- and double-strand DNA, respectively. As expected, we observed that RNase I/RNase A and RNase V1 treatments digest the EZH2 pulldown, whereas there are no effects with RNase H and DNase I treatments (Figure 1C). These results indicate that molecules in the EZH2-associated complex are single- and/or double-strand RNAs.

We generated 41,240,432 reads for EZH2 RIP samples and 32,117,284 reads for mock RIP samples. The raw reads were mapped against the human reference genome (GRCh37/hg19) using TopHat with default parameters [16]. After removing duplicate alignments and filtering out primer dimers, rRNA, and mitochondrial RNA, we obtained 3,086,721 unique reads from EZH2 libraries and only 2,226,630 unique reads in IgG libraries (Supplementary Table 1).

### The EZH2-associated transcriptome

**Figure 2:**
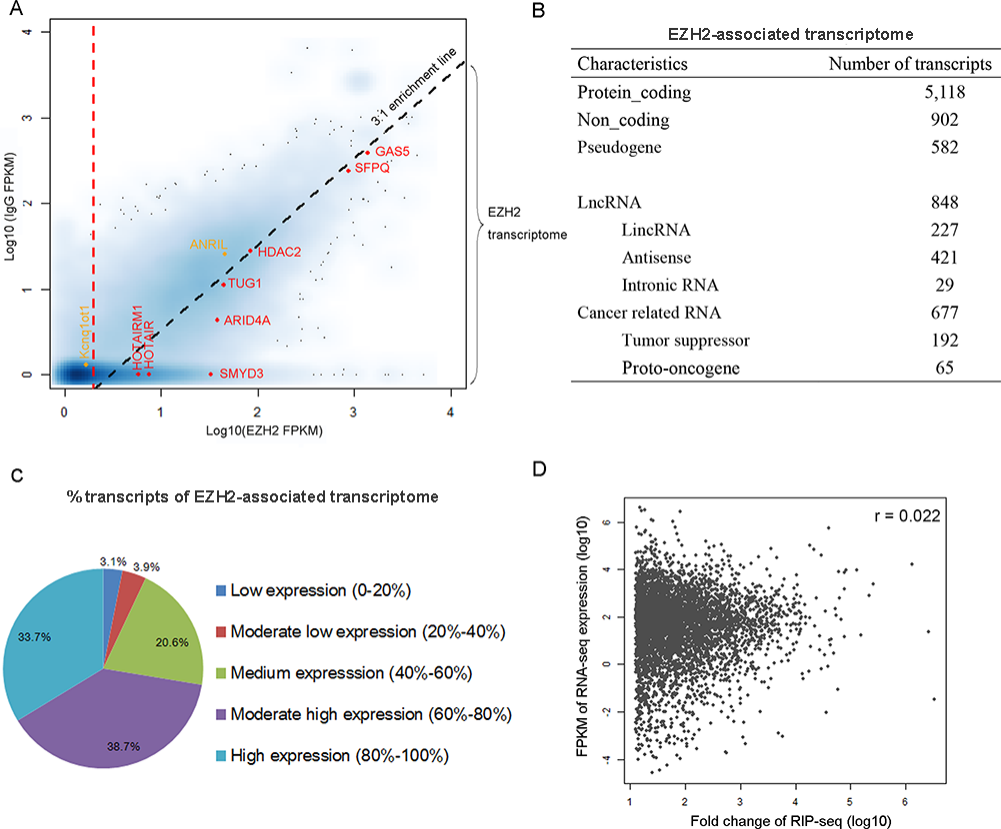
Characterization of EZH2-associated transcriptome in gastric cancer cells (**A**) The scatterplot maps transcripts by their FPKM values in the EZH2-RIP (x axis) and IgG-RIP (y axis). Smoothing was carried out by the function, smoothScatter, in R. Darker shades indicate genes with higher density on the graph. Diagonal dashed line represents the 3:1 EZH2/IgG enrichment threshold. Vertical dashed line represents a cutoff of FPKM [EZH2] = 2. Red, selected transcripts previously suggested to be associated with PRC2. Orange, selected transcripts of known EZH2-associated RNAs but excluded from the EZH2-associated transcriptome in MKN45. (**B**) Characteristics of the EZH2-associated transcriptome. (**C**) Composition of the populations of EZH2-associated RNAs expressed at various levels in gastric cancer cells. All genes were ranked by expression level. (**D**) Correlations between the expression level of RNAs and their enrichment in the EZH2-immunoprecipitated fractions (FC values).

To define a threshold for identifying transcripts that constitute the “EZH2-associated transcriptome”, we used a strategy similar to that described in a recent study [14] with the assumptions that the read density of the EZH2-interacting transcripts should be higher than the control and the positive transcripts should be enriched in the EZH2 pulldown relative to the control. We then calculated genic representation with “fragments per kilobase of transcript per million reads” (FPKM) for normalization of gene length and sequencing depth and mapped all transcripts in the Ensembl database to a scatter-plot by their EZH2 FPKM (x axis) and control IgG FPKM (y axis) values (Figure 2A). Transcripts with near-zero or zero representation in both libraries constitute the majority of data points (blue cloud at [0, 0]). Transcripts with a nonzero *x* value and a zero *y* value indicate a population represented only in the EZH2 pulldown. We then calculated fold change (FC) as the ratio of FPKM [EZH2] / FPKM [IgG] and performed RIP RT-qPCR validation to determine a threshold that enabled the identification of the EZH2-interacting RNAs against the control (Supplementary Figure 1A). We found that the criteria of FC > 3 and FPKM [EZH2] > 2 reliably distinguished the EZH2-associated transcripts from the control. With these criteria, we identified 8,256 RNAs that are associated with EZH2 in the three cell lines (Supplementary Table 2). We found that the majority of EZH2-associated RNAs are derived from MKN45 and the interactions of EZH2 and RNAs occur relatively less frequently in AGS and GES-1 (Supplementary Figure 2), probably due to the high expression of EZH2 in MKN45 [17].

EZH2-associated transcripts in MKN45 (Figure 2B) are distributed in both noncoding and protein-coding regions and transcribed from both strands. Among the identified transcripts we observed several well-characterized RNAs previously reported to be associated with PRC2, including HOTAIR [9], TUG1 [18], ARID4A, and SMYD3 [19] (Figure 2A). However, we observed that other recognized EZH2-bound RNAs, such as ANRIL [10] and Kcnq1ot1 [20], do not meet these criteria and thus were excluded from the EZH2-associated transcriptome in GC cells, probably due to the difference of cellular context.

To demonstrate the reliability of these EZH2-associated transcripts, we performed RIP assays under more stringent conditions by lengthening the washing times and using more stringent washing buffers. We randomly selected several transcripts and performed RT-qPCR validation. All show significant enrichment in the EZH2 pulldown relative to the IgG pulldown (Supplementary Figure 1B).

To rule out the possibility that the enrichment of EZH2-bound transcripts is due to their high expression, we compared RIP-seq data with the RNA-seq data in MKN45 generated in our recent report [17]. We found that a considerable number of EZH2-associated RNAs are expressed at low to medium levels (Figure 2C). Moreover, there is no correlation between expression level and enrichment (Figure 2D). Collectively, these results indicate that the EZH2-bound transcripts are highly specific.

### Genomic features of transcripts associated with EZH2

**Figure 3:**
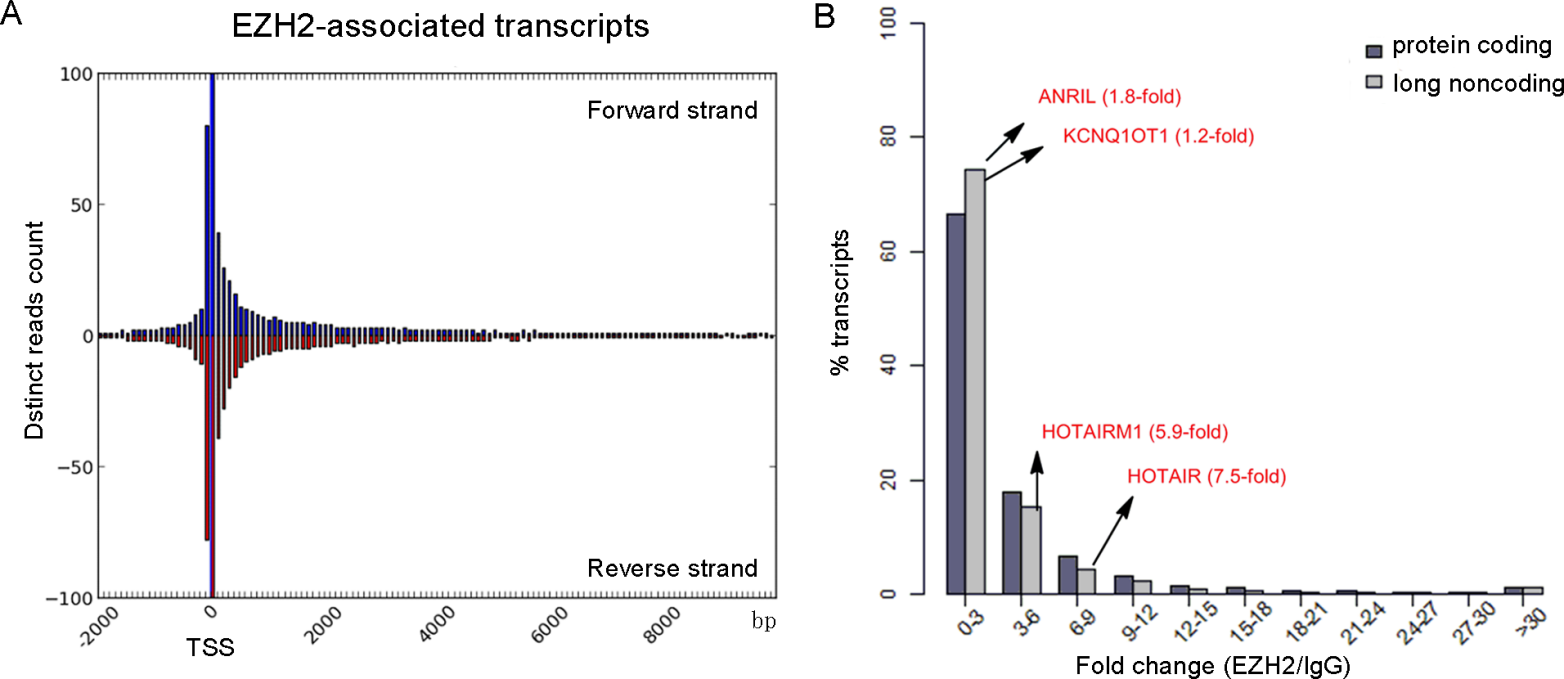
Genomic features of EZH2-associated transcriptome (**A**) Distinct reads from the EZH2-associated transcriptome were plotted as a function of distance from TSS. (**B**) Comparison of the binding capacity to EZH2 between protein-coding RNAs and long noncoding RNAs.

Recent studies have identified many promoter-associated RNAs that alter gene transcription through interaction with protein complexes [21]. To examine their genomic features, we queried the relationship of EZH2-associated transcripts to transcription start sites (TSSs) by plotting the read numbers as a function of genomic distance (Figure 3A, Supplementary Figure 3). We found an obvious enrichment of reads within the region of −0.5 to +1 kb from TSSs in both forward and reverse strands. The 5′ bias in the EZH2-RNA contacts is consistent with a previous study of embryonic stem cells [22], indicating that EZH2 binds the 5′ region of some transcripts.

A recent mRNA-proteome interaction study has revealed that mRNA molecules frequently interact with transcriptional regulators [23], suggesting a potential regulatory role of mRNA in gene expression. We found that 77.5% of EZH2-associated RNAs are protein-coding transcripts (Figure 2B). To compare the binding features of protein-coding and noncoding transcripts, we analyzed the binding affinity of EZH2-interacting transcripts in our data set with a reported method [13] and found similar enrichment levels between mRNAs and lncRNAs. Specifically, we observed that many coding transcripts bound with even stronger affinities to EZH2 than some well-known lncRNAs, including HOTAIR and HOTAIRM1 (Figure 3B). To explore the biological relevance of the EZH2-associated protein-coding RNAs with high read coverage (> 10 FPKM), we performed a gene ontology (GO) analysis. The most prominent GO in terms of biological process are shown in Supplementary Figure 4, including cell cycle and metabolic processes of DNA and RNA. This result suggests a potential negative regulatory role of the EZH2-associated coding transcripts.

We next examined the presence of long intergenic non-coding RNAs (lincRNAs) in our data set and observed 227 lincRNAs, accounting for 3.4% of the EZH2-associated transcriptome (Figure 2B). Additionally, we identified 421 (6.4%) antisense RNAs. The GO analysis indicates that genes associated with these antisense transcripts are involved in regulation of transcription, RNA metabolic process, histone modification, and the cell cycle (Supplementary Figure 5).

Among the 6,602 EZH2-interacting RNAs we found 677 (10.3%) are derived from cancer-related genes (http://www.bushmanlab.org/links/genelists), including 192 tumor suppressors [24] and 65 proto-oncogenes (http://www.uniprot.org/) (Figure 2B). These cancer-related genes are involved in MAPK, p53, and ERBB signaling pathways (Supplementary Table 3). These observations suggest that EZH2-bound RNAs potentially regulate the expression of cancer-related genes by interacting with PRC2 in gastric cancer cells.

### LncRNA MALAT1 tethers EZH2 and suppresses *PCDH10*

LncRNAs are emerging as important players in malignancy [25]. We compared the EZH2-bound transcripts among two gastric cancer cell lines and their normal counterpart. Among 848 EZH2-bound lncRNAs in MKN45, the metastatic cell line among the three cell lines examined, we found HOTAIR and MALAT1, which are well documented for their involvement in cancer metastasis [25, 26]. HOTAIR is not actively transcribed, whereas MALAT1 is highly expressed in MKN45 (Supplementary Table 4), suggesting its potential role in gastric malignancy.

MALAT1 interacts with EZH2 to suppress E-cadherin (*CDH1*) expression in renal cell carcinoma [27]. We found the expression of *CDH1* is less affected upon EZH2 knockdown in MKN45 [17], suggesting *CDH1* is not likely targeted by the MALAT1-EZH2 complex. To explore potential targets whose transcription is modulated by EZH2-MALAT1 interaction, we identified 954 up-regulated genes upon EZH2 knockdown with RNA-seq data obtained in our previous report [17]. Of these EZH2 responsive genes are 74 genes involved in cell adhesion, including 9 tumor suppressor genes (TSGene database: http://bioinfo.mc.vanderbilt.edu/TSGene/index.html) (Supplementary Table 4). Interestingly, we found protocadherin 10 (*PCDH10*) is one of these 9 tumor suppressor genes. Similar to *CDH1*, *PCDH10* inhibits cancer cell motility and migration [28]. *PCDH10* is targeted by EZH2 [29] and thus, we assumed that *PCDH10* is suppressed by MALAT1-mediated EZH2 silencing.

**Figure 4:**
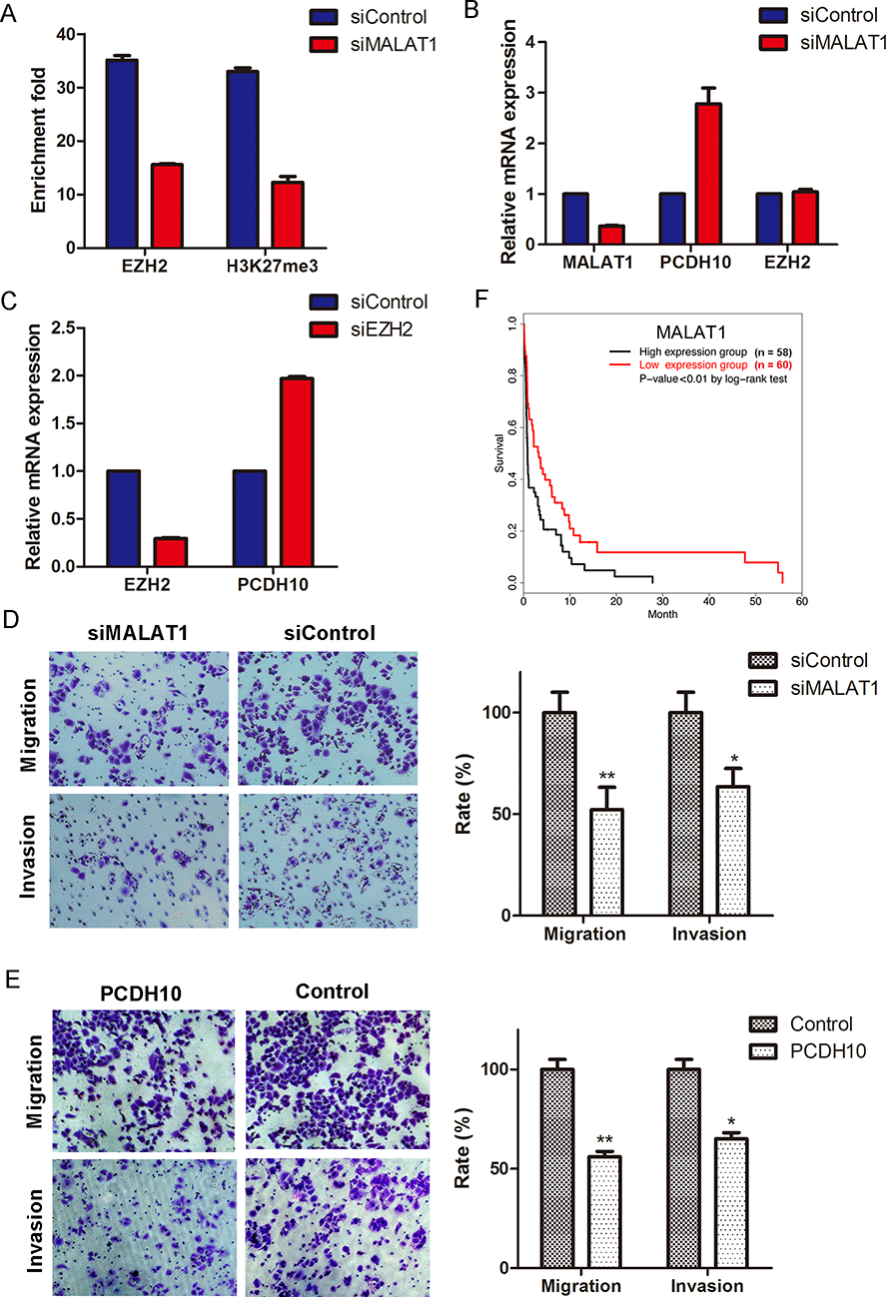
MALAT1 suppresses *PCDH10* by interacting with EZH2 and promotes gastric cancer cellular invasion and migration (**A**) ChIP-qPCR assay of the occupancy of EZH2 and H3K27me3 at *PCDH10* promoter in MALAT1-knockdown cells. (**B**) Expression measurement of *PCDH10* and *EZH2* by RT-qPCR in MALAT1-knockdown cells. (**C**) Expression measurement of *PCDH10* by RT-qPCR in EZH2-knockdown cells. The expression of mRNAs was normalized to *GAPDH* (means ± SEM, *n* = 3). (**D** and **E**) MALAT1 knockdown (D) and overexpression of PCDH10 (E) inhibit gastric cellular invasion and migration. Images show a representative field. Invasion and migration are expressed as a percentage of controls (means ± S.D., *n* = 3). Two-tailed Student's *t* test, *p < 0.05; **p < 0.01. (**F**) The association between stages III and IV gastric cancer patients’ survival and MALAT1 expression was estimated using the Kaplan–Meier method and the log-rank test (*p* < 0.01).

To address this assumption, we knocked down MALAT1 in MKN45 and observed a decrease in both H3K27me3 and EZH2 occupancy at the *PCDH10* promoter (Figure 4A). Meanwhile, we found *PCDH10* is obviously up-regulated upon MALAT1 knockdown (Figure 4B). In addition, we found the loss of EZH2 occupancy at the *PCDH10* promoter does not result from reduced EZH2 expression since there is no transcriptional alteration of EZH2 upon MALAT1 knockdown (Figure 4B). Finally, we observed the activation of *PCDH10* expression when *EZH2* was knocked down (Figure 4C). Taken together, these results indicate that MALAT1 is involved in suppressing *PCDH10* through interaction with EZH2.

### LncRNA MALAT1 promotes GC cellular migration and invasion and is associated with poor prognosis

To examine the biological significance of MALAT1 in GC cells, we performed transwell migration and invasion assays. Compared with the control, MALAT1 knockdown decreases invasion and migration of MKN45 by ∼ 64% and 52%, respectively (Figure 4D). Meanwhile, we found a similar phenomenon when *PCDH10* is overexpressed (Figure 4E).

In order to further examine the prognostic value of lncRNA MALAT1 in GC patients, we obtained RNA-seq data of 250 patients (157 male and 93 female) with various stages of cancer from The Cancer Genome Atlas (TCGA) database and performed Kaplan-Meier survival analysis based on the expression levels of MALAT1. We found that highly expressed MALAT1 is associated with poor overall survival among stage III and IV GC patients (*p*-value < 0.01 by log-rank test, Figure 4F), whereas such association is not significant in stage I and II GC patients (data not shown).

## DISCUSSION

An increasing number of lncRNA-protein interaction studies suggest that lncRNAs are actively involved in a wide variety of biological processes. Therefore, many efforts have been made to develop approaches for identifying RNA molecules associated with a protein of interest in a genome-wide manner [14, 30]. In this study, we modified a previous RIP-seq method to identify EZH2-associated RNAs in gastric cancer cells. Our method has advantages over previously reported methods. Microarray-based analysis of protein-associated RNAs relies on prior knowledge of probe the sequence [30, 31], whereas RIP-seq does not require such information. In addition, the original RIP-seq approach utilized a template switching strategy for the 2nd cDNA synthesis and it has been known that such strategy exhibits a bias with a preferential detection of RNA molecules with guanine in their 5′ terminal end [32]. By contrast, our method utilized adaptor ligation to prime the 2nd cDNA synthesis which circumvents that bias. As such, we provide a robust and unbiased approach to perform transcriptome-wide investigations of protein-associated RNAs.

As a histone methytransferase that generates H3K27me3 and induces a transcriptionally repressive chromatin state, PRC2 was typically found at repressive genomic regions. Although it is well documented that lncRNAs are associated with PRC2 and involved in gene silencing [33], we found the majority of EZH2-interacting transcripts in gastric cancer cells are mRNAs, some derived from actively transcribed genes. A similar phenomenon was observed in a previous study of murine embryonic stem cells [14]. We found no correlation between the expression level of the EZH2-interacting transcripts and their binding enrichment to EZH2 (Figure 2D), suggesting that the RNA association with EZH2 is not due to the high expression level. Given the promiscuous RNA binding property of PRC2 [12], the extensive presence of mRNAs associated with PRC2 raises a possibility that these mRNA molecules play a regulatory role in gene expression, rather than being intermediates in protein synthesis [13]. More recently, two E3 ubiquitin ligases (Trim25 and Trim71) were identified as RNA binding proteins in embryonic stem cells and found to be associated with mRNAs [34], revealing a link between protein-bound mRNAs and the ubiquitin pathway, a pathway that is important in maintaining embryonic stem cell identity [35]. For gastric cancer cells it remains to be elucidated how mRNAs exert the regulatory activity through the interaction with PRC2.

**Figure 5:**
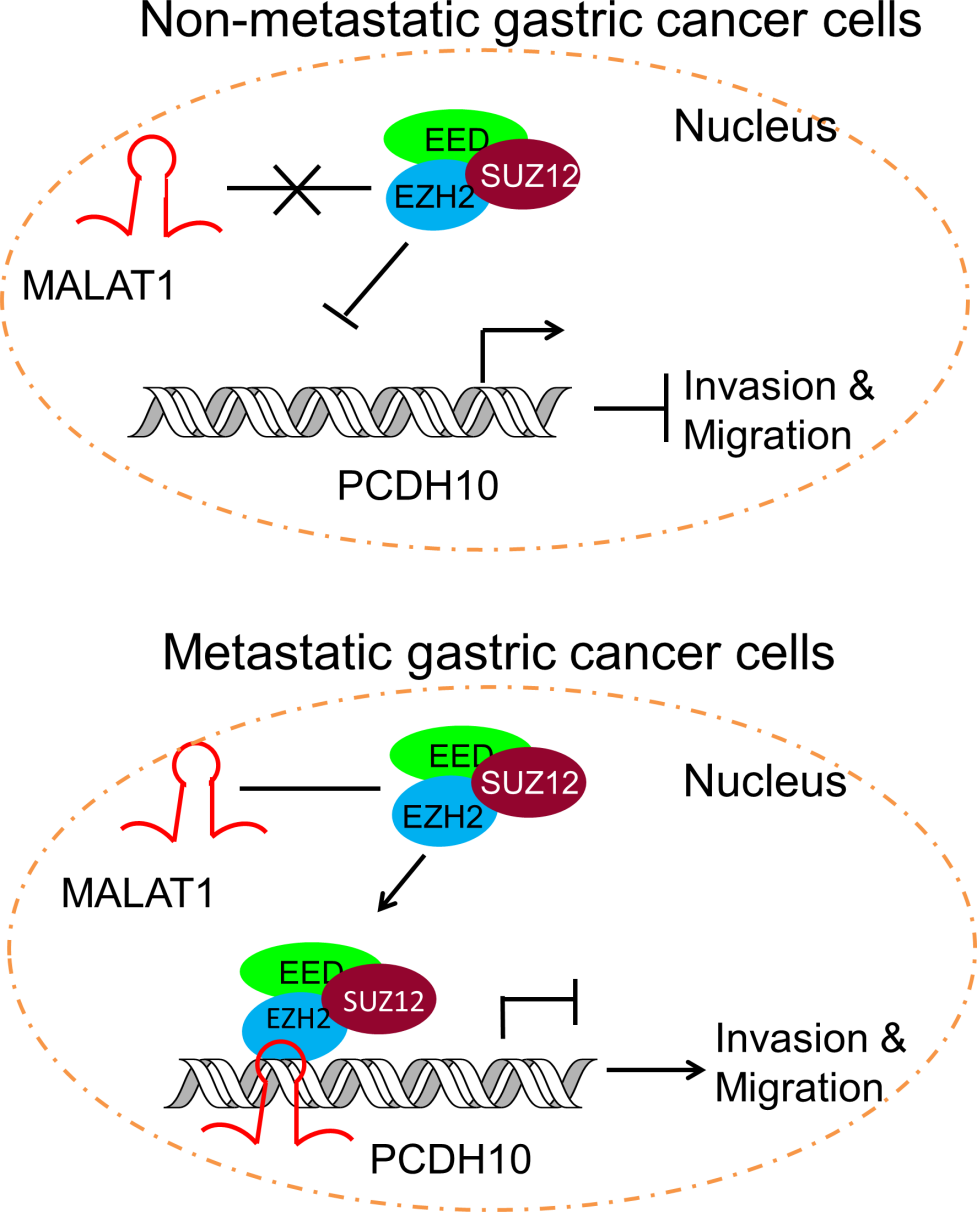
Schematic representation of the consequence of EZH2-MALAT1 interaction on the gastric cancer migration and invasion EZH2 is tethered by MALAT1 to *PCDH10* locus and promotes gastric cancer metastasis by suppressing *PCDH10*.

Long ncRNAs are rapidly emerging as new players in cancer biology [25, 36]. These lncRNAs contribute to cancer hallmark characteristics and operate transcriptionally, post transcriptionally, or epigenetically to regulate gene expression. Among the well documented lncRNAs involved in cancer migration and metastasis are HOTAIR and MALAT1 [25]. Both have been demonstrated to interact with and recruiting PRC2 for transcriptional silencing [9, 27]. In our study, we found HOTAIR is not transcriptionally active in MKN45, but MALAT1 is highly expressed. Although also highly transcribed in AGS, MALAT1 interacts with EZH2 only in the metastatic cell line MKN45 and such interaction is absent in both less-metastatic AGS cells and the normal counterpart GES-1 (Supplementary Table 2), suggesting a potential role of MALAT1 in gastric metastasis. MALAT1 is involved in metastasis through diverse mechanisms in various types of cancer [37, 38]. Here, we demonstrate that MALAT1 recruits EZH2 to the *PCDH10* promoter region and suppresses its transcription (Figure 4). Based on these observations, we proposed a model in which lncRNA MATLAT1 represses *PCDH10* and contributes to gastric cancer invasion and migration by recruiting PRC2 (Figure 5).

Briefly, we performed a transcriptome-wide EZH2-RNAs interaction analysis in gastric cancer cells with a modified RIP-seq approach. Our results reveal that the majority of EZH2-associated RNAs are mRNAs, together with a considerable fraction of ncRNAs. We demonstrate that the lncRNA MALAT1 recruits EZH2 to repress *PCDH10* and promotes gastric cancer metastasis. This study could serve as a valuable resource for understanding EZH2 biology and provides an insight into uncovering the role of lncRNA in tumorigenesis.

## MATERIALS AND METHODS

### Cell culture and RIP-seq

Gastric cancer cell lines MKN45 and AGS and the immortalized gastric epithelial cell line GES-1 were reported in our previous study [17] and maintained in RPMI-1640 (Gibco) supplemented with 10% heat-inactivated fetal bovine serum (Gibco) in an atmosphere of 5% CO_2_ at 37°C.

RNA immunoprecipitation was carried out as previously described with some modifications [14]. Briefly, nuclei from 10^7^ cells were isolated, and nuclear lysates were prepared, treated with 400 U/ml RNase-free DNase (Fermentas), and incubated with EZH2 antibodies (Active Motif) or control IgG (Cell Signaling Technology) overnight followed by stringent washing of protein A/G beads (Millipore). The pellets were finally resuspended in 1 ml Trizol (Invitrogen). To avoid DNA contamination, we treated RIP pull-downs with RNase-free DNase (Fermentas) according to the manufacturer's instructions. The 1st strand cDNA was synthesized with random primer flanked by a 7 nt sequence (Adaptor 1, Supplementary Table 5) to track strand information. The 2nd strand DNA was generated by the strategy we previously reported [15] with N6 adaptor (Adaptor 2, Supplementary Table 5). The resulting DNA libraries were amplified with Phusion^®^ High-Fidelity DNA Polymerase (NEB) as follows: 30 sec at 98°C, followed by a 4 cycles of (10 sec at 98°C, 10 sec at 66°C, 30 sec at 72°C) and 14–20 cycles of (10 sec at 98°C, 10 sec at 69°C, 30 sec at 72°C), additional extension for 10 min at 72°C and then hold at 10°C. PCR products were loaded on 2% agarose gel for size selection and 350–1500 bp products were excised and extracted by QIAquick Gel Extraction Kit (QIAGEN). DNA concentrations were quantitated by PicoGreen. cDNA samples were templates for Paired-ends Solexa sequencing on Illumina HiSeq2000 platform.

### Bioinformatics analysis

For the RIP-seq raw data, the sequencing reads matching the mitochondrial genome and ribosomal RNAs were excluded and the remaining reads were aligned to the Ensemble human genome GRCh37 using TopHat [16]. The default parameters were used. The human genome and corresponding annotation were obtained from Illumina's iGenomes project (http://support.illumina.com/sequencing/sequencing_software/igenome.ilmn). Cufflinks produced a list of identified transcripts with corresponding FPKM values, which allows us to compare samples with different sequencing depths and the transcripts with different lengths.

To generate promoter maps, we defined promoter regions as −10,000 to +2,000 bases relative to TSS which was obtained from Refseq Gene catalog in UCSC Genome Browse. We plotted read counts located at promoter regions.

To determine the biological functions of EZH2-associated RNAs, we performed a gene ontology analysis using DAVID (http://david.abcc.ncifcrf.gov/) with default parameters [39].

### RIP-qPCR

RIP was followed by quantitative real-time PCR using an ABI 7900 Real Time PCR Instrument, using the SYBR green PCR Master Mix (DBI bioscience). U1 was used as a control RNA for normalization. The primer sequences are shown in Supplementary Table 5.

To test the authenticity of EZH2-interacting RNAs, RIP assay under more stringent condition was performed [30] and RIP enriched RNAs were subjected to RT-qPCR. The primer sequences are shown in Supplementary Table 5.

### RNA interference and RT-qPCR

RNA interference assays were performed as we reported previously [17]. The siRNA targeting EZH2, MALAT1 and non-targeting control were synthesized by Shanghai GenePharma. The following *EZH2*- and MALAT1-targeting siRNA were used: 5′-GACUCUGAAUGCAGUUGCU-3′ and 5′-CGCAUUUACUAAACGCAGA-3′, respectively. Cells were transfected with siRNA or control siRNA using Lipofectamin^™^ 2000 Transfection Reagent (Invitrogen) according to the manufacturer's protocol.

Total RNA was extracted with TRIzol Reagent (Invitrogen) and resuspended in RNase free water. Reverse transcription of 1 μg RNA was performed using the oligo-dT primer and SuperScript^®^III Reverse Transcriptase (Invitrogen) according to the manufacturer's protocol. Expression levels were determined by real-time PCR with ABI step one plus (Applied Biosystems, USA). *GAPDH* was used as a control gene for normalization. The relative level of mRNA was calculated as 2^−ΔΔCt^. The primer sequences are shown in Supplementary Table 5.

### ChIP-qPCR

ChIP experiments for EZH2, H3K27me3, and IgG were carried out as we described previously [40]. Briefly, cells were cross-linked with 1% formaldehyde for 10 min at room temperature and formaldehyde was then inactivated by the addition of 125 mM glycine. Sonicated DNA fragments with 150–300 bp were immunoprecipitated using anti-EZH2 (Active Motif), anti-H3K27me3 (Millipore), and anti-IgG (Cell Signaling Technology) antibodies. The primer sequences are shown in Supplementary Table 5. The relative enrichment was calculated by determining the apparent immunoprecipitation efficiency (ratios of the amount of immunoprecipitated DNA over that of the input sample) and normalized to the level of GAPDH observed at a control region, which was defined as 1.0. The relative enrichment level was calculated as 2^−ΔΔCt^.

### Cell invasion and migration assays

Transwell assays were performed as we described previously [17]. Briefly, transwell filters (BD Biosciences) of 8 μm pore size were used. Full length *PCDH10* cDNA was cloned into pIRESneo3 for transwell assay. For invasion assay, transwell membranes were coated with Matrigel^™^ Basement Membrane (BD Biosciences). Transfected MKN45 cells were re-seeded onto the upper precoated chamber (2 × 10^4^ cells per well) in 100 μl of serum-free medium. Lower wells of the chamber contained 0.6 ml of 15% FBS-containing medium. Cells were incubated for another 48 h at 37°C in 5% CO_2_.

For cell migration assay, transfected MKN45 cells were re-seeded onto the upper chamber in 100 μl of serum-free medium (5 × 10^4^ cells per well). To the lower chambers, migration-inducing medium (with 10% FBS) was added. Then, cells were incubated for another 24 h at 37°C in 5% CO_2_.

### Statistical analysis

All *p*-values were calculated using two-tailed Student's *t*-test. A *p*-value < 0.05 was considered statistically significant. All experiments had at least two replicates.

### Data access

All raw sequencing files are available from the EMBL ArrayExpress database under accession number E-MTAB-3767.

## SUPPLEMENTARY MATERIALS FIGURES AND TABLES








