# Supplementary material for: MALAT1 long ncRNA promotes gastric cancer metastasis by suppressing PCDH10
Source: Oncotarget. 2016 Feb 9;7(11):12693–703. doi: 10.18632/oncotarget.7281 (PMC4914315; doi:10.18632/oncotarget.7281)
Supplement: Supplementary file 1 [file oncotarget-07-12693-s001.pdf]

MALAT1 long ncRNA promotes gastric cancer metastasis by suppressing *PCDH10*

Supplementary Materials

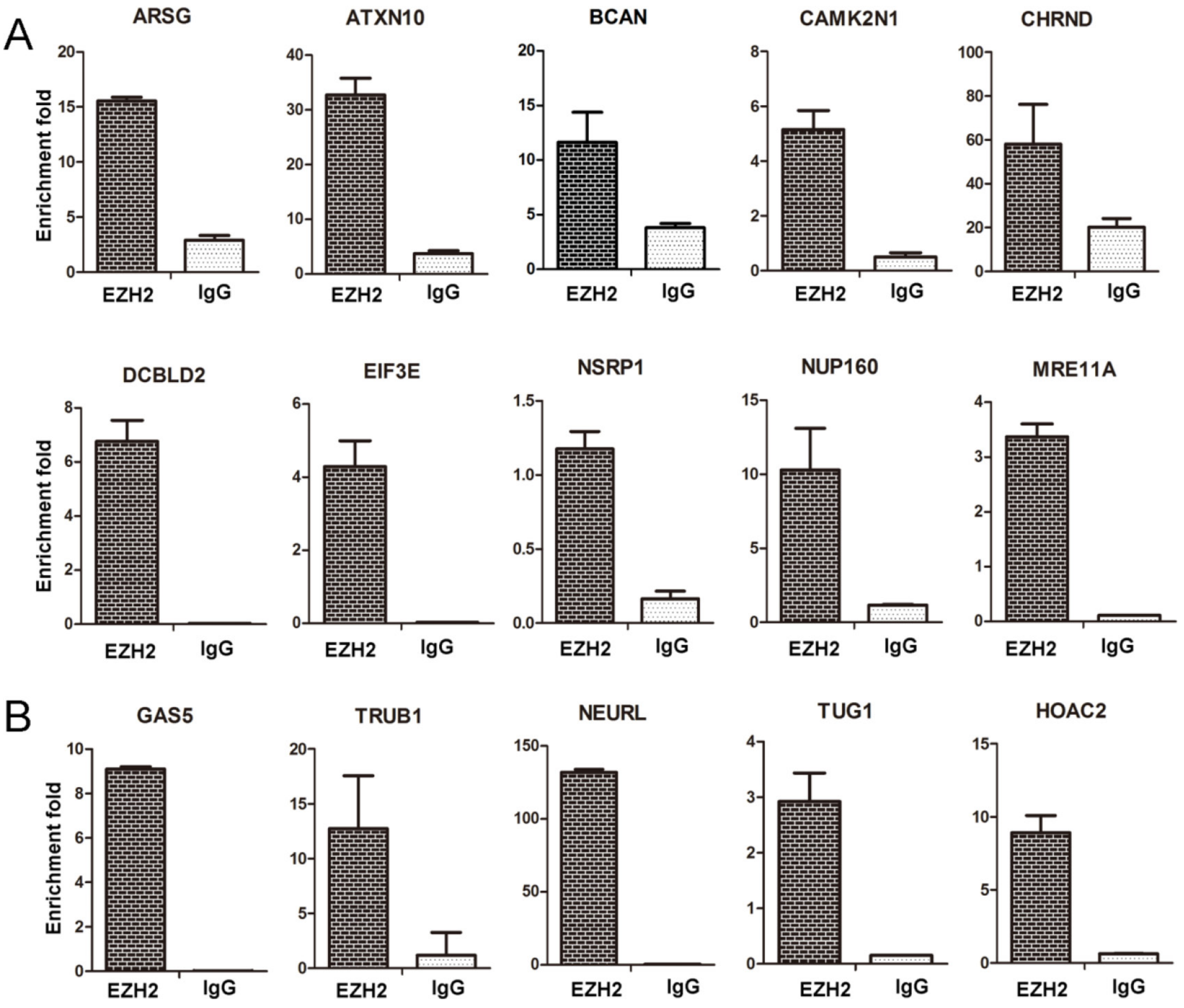

Supplementary Figure S1: RIP-qPCR validation. (**A**) Comparison of EZH2 and IgG pulldowns for several selected transcripts around the threshold by native RIP and RT-qPCR. (**B**) Validation of EZH2-bound transcripts captured by RIP under more stringent conditions with RT-qPCR.

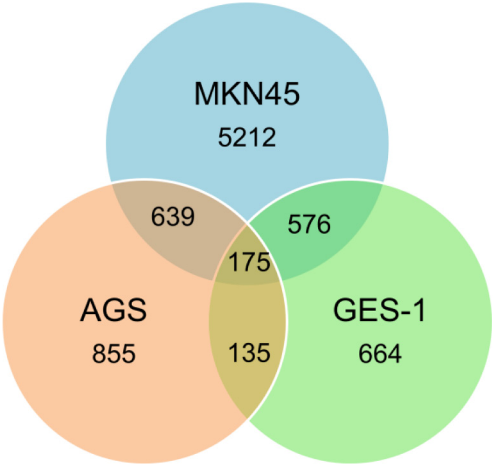

Supplementary Figure S2: A Venn diagram of the number of EZH2-bound transcripts identified in three cell lines.

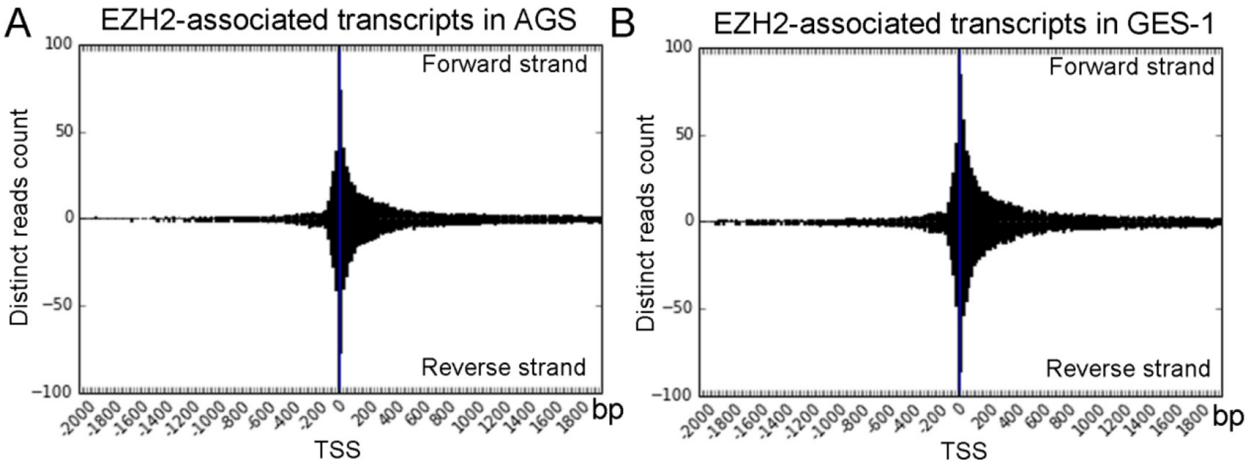

Supplementary Figure S3: Distinct reads from the EZH2-associated transcripts were plotted as a function of distance from TSS in AGS (A) and GES-1 cells (B).

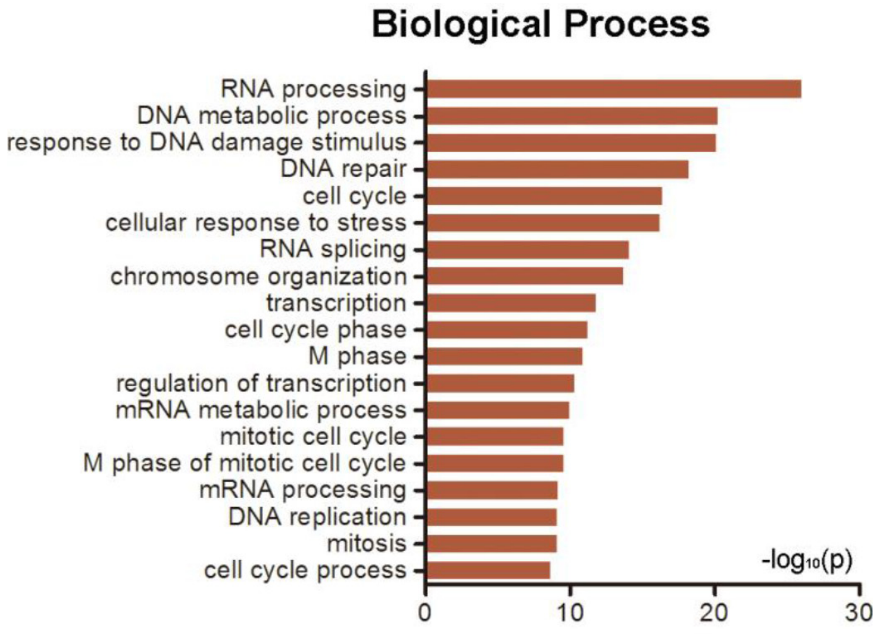

Supplementary Figure S4: Functional annotation of EZH2-associated mRNAs.

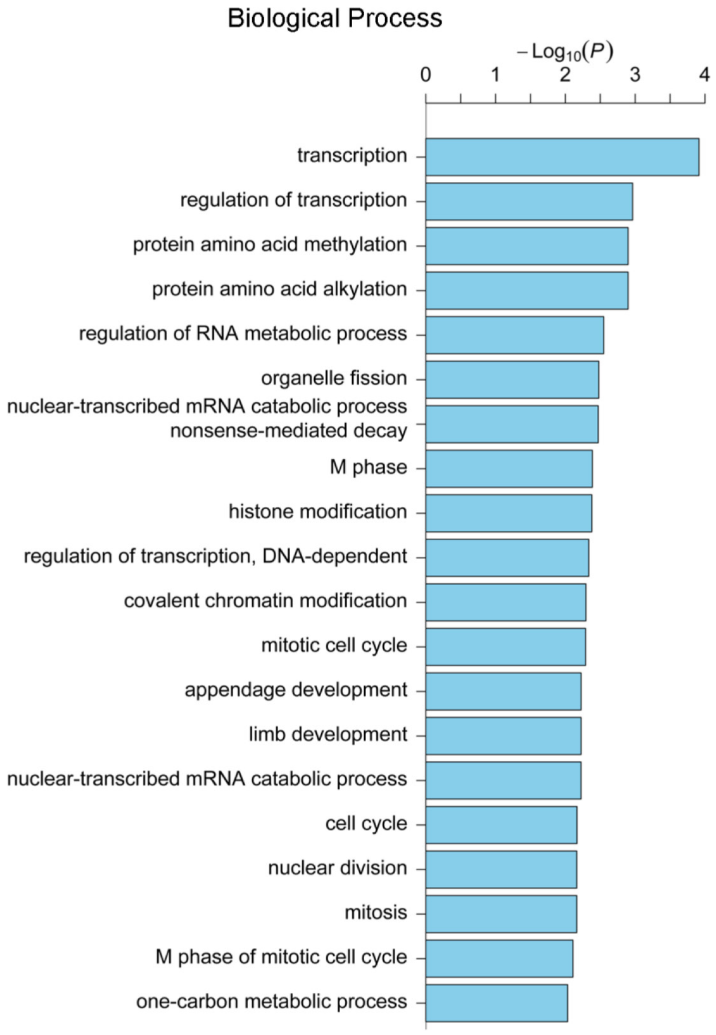

Supplementary Figure S5: A selection of the top enriched GO items for EZH2-interacting antisense transcripts.

Supplementary Table S1: Mapping statistics of RIP-seq data sets

Library	Total reads	Reads remaining after filtering	Total distinct reads	Number of reads		
				ncRNA	protein_coding	pseudogene
GES-1 EZH2	14343655	5288748	1277042	47459	311418	7183
GES-1 IgG	8712659	6463507	541162	14358	103734	7306
AGS EZH2	11674023	9799354	708808	10754	227219	2393
AGS IgG	14373546	12439745	1241987	15521	198640	6940
MKN45 EZH2	15222754	13842164	1100871	14888	151480	5342
MKN45 IgG	9031079	6272332	443481	5423	50807	688

Supplementary Table S2: EZH2-associated transcriptome in different cell lines

Supplementary Table S3: GO analysis of EZH2- interacting transcripts derived from cancer-related genes

Supplementary Table S4: RNA-seq analysis in MKN45

Supplementary Table S5: The lists of primers used in this study in adaptors of cDNA library

Name	Sequence (5′–3′)	
Adaptor 1	GTGCTCTTCCGATCTAGAGCANNNNNNNNN	
Adaptor 2	ACCGAGATCTACACTCTTTCCCTACACGACGCTCTTCCGATCTNNNNNN<br>AGATCGGAAGAGCGTCGTGTAGGGAAAGAGTGTAGATCTCGGT	top<br>bot

RIP-qPCR primers

Name	Sequence (5′–3′)
U1-F	ATACTTACCTGGCAGGGGAG
U1-R	CAGGGGGAAAGCGCGAACGCA
MRE11A-F	CTCTCACCCAGACCCACCTA
MRE11A-R	GATGCTGTGCTACCTACCCC
CHRND-F	CCCCTACTCCTACAACGTGC
CHRND-R	CTCCTCTCTCAGACCCTGCT
NSRP1-F	GTCCATGCCTGTAGTCCCAG
NSRP1-R	TGGCATGATCATACCTCACTGT
EIF3E-F	CCCCTCACCTCCTTTACAGAG
EIF3E-R	GACTTGACTACTCGCATCGC
NUP160-F	CGCCCTCCTATCTTGTGCAG
NUP160-R	GGAACCCTGTACTCAAGGCC
ATXN10-F	GTTAGGGCTGTGTAGGGCG
ATXN10-R	GAAGGCGAGGAGGACGAAG
ARSG-F	ACAAGAGGACAGAAGCCAAACT
ARSG-R	AGGTTGGCAGTGTCCTTTGT
BCAN-F	CATGTCCCTCTGTCAGCCTG
BCAN-R	AGCTGTCTCCTTCCAGAACA

CAMK2N1-F	ATCTGTCTCCCGGCCTGATA
CAMK2N1-R	CGGCGGTAACAGTTATTGGC
DCBLD2-F	GGTTCCCCGTAGCCTTGAAA
DCBLD2-R	GCCTGAGTATGCAACCCCAA
GAS5-F	TCGTACTGACGAAGGTGCAT
GAS5-R	AGATGGGCGTCACTACTGCT
TRUB1-F	GCTACCAAGCTGCTGTCCTT
TRUB1-R	TCTCCTTCAACCGATTCAGC
HDAC2-F	GAAAAGGGCTGAGGGAAACG
HDAC2-R	GTGGTACCGAGCCTTCCC
TUG1-F	GTCTTTGACCCCAGGAATGA
TUG1-R	TCCCATTCTAGGAATCACTGG
NEURL-F	ACAGGGAGACCTTGTGATGG
NEURL-R	GAACCTGGAGGTGTGAGAGG

ChIP-qPCR primers

Name	Sequence (5′–3′)
GAPDH-F	AAGACCTTGGGCTGGGACT
GAPDH-R	GCTGCGGGCTCAATTTATAG
PCDH10-F for anti-EZH2	GGCTTGGGAAGAAAACAGCA
PCDH10-R for anti-EZH2	TCCCTCTGTCCTCATCTCCA
PCDH10-F for anti-H3K27me3	TCCGCCTCAAGTCTTCCTTT
PCDH10-R for anti-H3K27me3	CAGTCAGGGTGTAGGAGTCC

RNAi-qRT-PCR primer

Name	Sequence (5′–3′)
EZH2 mRNA-F	TTGTTGGCGGAAGCGTGTAAAATC
EZH2 mRNA-R	TCCCTAGTCCCGCGCAATGAGC
GAPDH mRNA-F	GCACCGTCAAGGCTGAGAAC
GAPDH mRNA-R	ATGGTGGTGAAGACGCCAGT
MALAT1 mRNA-F	AAAGCAAGGTCTCCCCACAAG
MALAT1 mRNA-R	GGTCTGTGCTAGATCAAAAGGCA
PCDH10-mRNA-F	CAGACCTCGCCGAGTTAACA
PCDH10-mRNA-R	ACGGTTGGTGGCATCATGAT
